# Microsurgical resection of unruptured cerebellar arteriovenous malformation presenting with trigeminal neuralgia

**DOI:** 10.3171/2020.10.FOCVID2071

**Published:** 2021-01-01

**Authors:** Walter Marani, Nicola Montemurro, Shoichiro Tsuji, Paolo Perrini, Kosumo Noda, Nakao Ota, Yu Kinoshita, Hiroyasu Kamiyama, Rokuya Tanikawa

**Affiliations:** 1 Far East Neurosurgical Institute, Sapporo Teishinkai Hospital, Sapporo, Japan;; 2 Department of Neurosurgery, Azienda Ospedaliera Universitaria Pisana (AOUP), Pisa; and; 3 Department of Translational Research on New Surgical and Medical Technologies, University of Pisa, Italy

**Keywords:** posterior fossa, arteriovenous malformation, AVM, surgery, surgical treatment

## Abstract

Cerebellar arteriovenous malformations (AVMs) represent 10%–15% of all intracranial AVMs and are associated with a greater risk for hemorrhagic presentation compared with supratentorial AVMs. When they reach the cerebellopontine angle cistern, neurovascular compression syndromes, including trigeminal neuralgia and hemifacial spasm, can occur. Due to the aggressive natural history of cerebellar AVM, an effective treatment strategy is required. In this video, the authors demonstrate the technical nuances of microsurgical resection of an unruptured cerebellar AVM in a 24-year-old female presenting with trigeminal neuralgia. The patient underwent right retrosigmoid craniotomy and complete resection of the AVM with resolution of trigeminal neuralgia.

The video can be found here: https://youtu.be/6GmNjgFQwx8

## Transcript

**0:20 Clinical Presentation.** In this video, we will discuss the surgical treatment of posterior fossa arteriovenous malformation (AVM) in a young patient. The relevant anatomy and technical nuances will also be presented. This is a 24-year-old woman with right trigeminal neuralgia and right upper-limb ataxia associated with a Spetzler-Martin^1^ grade III unruptured AVM located in the right cerebellopontine angle (CPA). The trigeminal neuralgia did not improve after medical treatment. Patient refused radiosurgery and endovascular treatment and chose for surgical removal of the AVM.^2^

**0:55 Preoperative Imaging.** The preoperative angiogram demonstrated the main feeders coming from the superior cerebellar artery (SCA), anterior inferior cerebellar artery (AICA), and from the posterior inferior cerebellar artery (PICA). The venous drainage was in the right Rosenthal vein (through the right superior hemispheric vein), in the superior petrosal sinus (through the superior petrosal vein), and in the transverse sinus (through the inferior vermian vein). No venous outflow stenosis nor flow-related aneurysms were demonstrated on preoperative DSA. The MRI with FIESTA sequences was useful to assess the relationship between the vascular structures and the cranial nerves, showing the compression and distortion of the right trigeminal nerve by the SCA. Reconstruction of the 4D computed tomography (CT) angiogram were used to assess the relationship with bony structures and planning the craniotomy. The surgical plan was to achieve early proximal control through temporary clipping of the main arterial feeders during subarachnoid dissection and to start the subpial dissection with reduced blood flow in the draining veins.

**2:10 Patient Position and Craniotomy.** The patient was positioned in a park-bench position with the head fixed in a three-pin frame. The skin and subcutaneous tissues opening was performed in a layer-by-layer fashion until the asterion, digastric incisura, and condylar fossa were exposed. A right retrosigmoid craniotomy was performed in a standard fashion exposing the transverse-sigmoid junction and was enhanced by skeletonizing the entire sigmoid sinus. After craniotomy, the posteromedial surface of the occipital condyle was drilled until the exposure of the hypoglossal canal. Finally, the hypoglossal nerve was confirmed through direct electrical stimulation.

**3:00 AVM Dissection.** The dura mater was opened in a C-fashion and reflected on the sigmoid sinus. It is possible to appreciate the superior hemispheric draining vein on the suboccipital surface of the cerebellum. The subarachnoid dissection was started to identify the main feeders and the cranial nerves.^3,4^ The subarachnoid dissection is of paramount importance for distinguishing the arterial and venous structures related with the AVM from the normal vessels, which should be preserved. At first, the fifth nerve was exposed, which was compressed and distorted by the right SCA.^5^

We perform the subarachnoid dissection under the highest magnification, which allows the identification of the correct plane of dissection. Sharp arachnoid dissection prevents the stretching and avulsion of the vascular structures and therefore minimizes complications during early stages of AVM surgery. The AICA was dissected and exposed. It was in close relationship with the seventh and eighth nerves. The facial nerve was confirmed through direct electrical stimulation. The intraoperative indocyanine green (ICG) angiogram showed the arterialized vein structures. Finally, the lower cranial nerves and PICA were dissected. The choroid plexus, protruding from the foramen of Luschka, is well visualized above the origin of the ninth cranial nerve. The sixth cranial nerve was also identified in its cisternal portion, going toward Dorello’s canal.

**5:05 AVM Removal.** After the subarachnoid dissection and identification of the arteries, the main feeders were occluded with temporary clips in this order: SCA, PICA, and AICA. We perform temporary clipping in the early phase of AVM surgery because it allows the repositioning of the clip during the following steps of dissection, avoiding ischemia in the territory of a temporary occluded “en passage” artery. In our experience, temporary proximal artery clipping helps in safe AVM dissection by reducing intraoperative bleeding and resection time. The PICA was followed in its route to the tonsillar vallecula and then clipped, avoiding the occlusion of branches not related to the AVM. Finally, the AICA, which was directly feeding the AVM, was occluded. Afterwards, the ICG showed a reduction in the draining veins’ flow. After the exclusion of the main feeders, the subpial dissection was started. The subpial dissection was carried circumferentially around the AVM, disconnecting the malformation from the surrounding parenchyma. The bleeding from the deep perforators was controlled with bipolar coagulation and a single clip. An accurate arachnoid dissection allowed us to minimize the subpial dissection and therefore the transgression of normal cerebellar tissue. At the end of the pial dissection, the connections with the draining veins were coagulated and cut; then the AVM was resected. Finally, the drainage toward the superior petrosal vein was coagulated and cut. The last ICG showed no residual AVM with no arterialized veins. You can well appreciate how even the ectasic venous structures fill during the venous phase of the ICG. The dura mater was closed in a watertight fashion with an autologous graft.

**9:30 Postoperative Course.** The postoperative 4D CT angiogram showed no remnants. The postoperative DWI MRI showed no hyperintensities signs. The patient woke up without any deficits and was discharged at home with modified Rankin score (mRS) 0 with full recovery of trigeminal neuralgia at the last follow-up.

## Author Contributions

Primary surgeon: Tanikawa. Assistant surgeon: Tsuji, Noda, Kinoshita. Editing and drafting the video and abstract: Marani, Montemurro, Tsuji. Critically revising the work: Marani, Montemurro, Perrini, Kinoshita, Tanikawa. Reviewed submitted version of the work: Marani, Montemurro, Perrini, Ota, Tanikawa. Approved the final version of the work on behalf of all authors: Marani. Supervision: Kamiyama, Tanikawa.
